# Novel antiviral activity of mung bean sprouts against respiratory syncytial virus and herpes simplex virus −1: an *in vitro* study on virally infected Vero and MRC-5 cell lines

**DOI:** 10.1186/s12906-015-0688-2

**Published:** 2015-06-11

**Authors:** Rand R Hafidh, Ahmed S Abdulamir, Fatimah Abu Bakar, Zamberi Sekawi, Fatemeh Jahansheri, Farid Azizi Jalilian

**Affiliations:** Department of Microbiology, College of Medicine, Baghdad University, Baghdad, Iraq; Institute of Bioscience, University Putra Malaysia, Serdang, 43400 Selangor Malaysia; Department of Microbiology, College of Medicine, Alnahrain University, PO Box 70030, Baghdad, Iraq; Faculty of Food Science and Technology, University Putra Malaysia, Serdang, 43400 Selangor Malaysia; Faculty of Health Sciences and Medicine, University Putra Malaysia, Serdang, 43400 Selangor Malaysia; Department of Medical Microbiology, Faculty of Medicine,, Hamadan University of Medical Sciences (HUMS), Hamadan, Ilam Iran

**Keywords:** Mung bean sprout, Respiratory syncytial virus, Herpes simplex virus-1, Ribavarin, Acyclovir, Cytotoxicity assay, Interferon, Tumor necrosis factor

## Abstract

**Background:**

New sources for discovering novel antiviral agents are desperately needed. The current antiviral products are both expensive and not very effective.

**Methods:**

The antiviral activity of methanol extract of mung bean sprouts (MBS), compared to Ribavarin and Acyclovir, on respiratory syncytial virus (RSV) and Herpes Simplex virus −1 (HSV-1) was investigated using cytotoxicity, virus yield reduction, virucidal activity, and prophylactic activity assays on Vero and MRC-5 cell lines. Moreover, the level of antiviral cytokines, IFNβ, TNFα, IL-1, and IL-6 was assessed in MBS-treated, virally infected, virally infected MBS-treated, and control groups of MRC-5 cells using ELISA.

**Results:**

MBS extract showed reduction factors (RF) 2.2 × 10 and 0.5 × 10^2^ for RSV and HSV-1, respectively. The 2 h incubation virucidal and prophylactic selectivity indices (SI) of MBS on RSV were 14.18 and 12.82 versus Ribavarin SI of 23.39 and 21.95, respectively, and on HSV-1, SI were 18.23 and 10.9 versus Acyclovir, 22.56 and 15.04, respectively. All SI values were >10 indicating that MBS has a good direct antiviral and prophylactic activities on both RSV and HSV-1. Moreover, interestingly, MBS extract induced vigorously IFNβ, TNFα, IL-1, and IL-6 cytokines in MRC-5 infected-treated group far more than other groups (*P* < 0.05) and induced TNFα and IL-6 in treated group more than infected group (*P* < 0.05).

**Conclusions:**

MBS extract has potent antiviral and to a lesser extent, prophylactic activities against both RSV and HSV-1, and in case of HSV-1, these activities were comparable to Acyclovir. Part of the underlying mechanism(s) of these activities is attributed to MBS potential to remarkably induce antiviral cytokines in human cells. Hence, we infer that MBS methanol extract could be used as such or as purified active component in protecting and treating RSV and HSV-1 infections. More studies are needed to pinpoint the exact active components responsible for the MBS antiviral activities.

## Background

During the past decade, many potent agents have become available against viral infections; however, the increasing clinical use or the abuse of these agents has been associated with the emergence of dangerous drug-resistant viral strains. In addition, the dose-limiting toxic effects of the known antiviral agents have been observed among patients especially immunocompromised individuals [1–3].

Plant bioactive molecules which occur in plants as secondary metabolites have significant defense mechanisms against predation, herbivores, fungal attack, microbial invasion, and viral infections [4]. Therefore, during the recent decade, extracts of plants as phytochemicals, such as phenolic compounds derived from the secondary plant metabolism, are getting more important as potential sources for viral inhibitors. Many studies showed a great range of pharmacological effects of these substances, including vasodilatation, antiallergenic, anti-inflammatory and antiviral properties [5]. More to the point, the relative success of various species of medicinal plant extracts with antiviral properties has raised optimism about the future of phyto-antiviral agents [6].

The discovery of safe and effective antiviral agents from plant extracts may secure humanity from the drug resistant viruses. Therefore, in order to discover new sources of safe antiviral therapies, the objectives of the current study are to investigate whether there is any antiviral activity of mung bean sprout (*Vigna radiata* L.), or (MBS), methanol crude extract and to assess part of the underlying mechanism of action of the antiviral activity, if any, of this methanol crude extract. Discovering effective antiviral plant extract is important in breaking the long-lasting shortage of antiviral drugs in industry and to boost the safety use of antiviral agents. MBS antiviral activity can be used in the form of extract or by isolating the responsible active component(s).

## Results

To investigate the *in vitro* antiviral properties of MBS extract, four approaches were performed. These methods included end point titration technique (EPTT), plaque assay, cytopathic reduction assay, and microculture tetrazolium assay (MTT).

### Estimating the antiviral activity by virus yield reduction assay

It has been shown that the virus yield reduction assay is a powerful technique for evaluating the efficacy of potential antiviral compounds [7]. In order to assess the antiviral activity, the maximum nontoxic dose of MBS extract, *i.e.*, IC50 was applied to virus host cells after infection with certain virus in an attempt to measure the reduction of virus titer. The reductions of virus titers (RSV and HSV-1) were determined in term of virus titer log after treatment in addition to the reduction factor (RF), *i.e.* the ratio of the virus titer in the absence of the extract over virus titer in the presence of the extract [8].

In this study, MBS extract showed moderate antiviral activity on HSV-1 titration in which HSV-1 titer was reduced by two log (Table 1). On the other hand, MBS extract showed slight antiviral activity against RSV as RSV virus titer was reduced by one log (Table 1). MBS extract showed RF values of ≥ 10 indicating a pronounced antiviral activities.

**Table 1:** The reduction in the RSV and HSV-1 titers after MBS extract treatment. The virus titer was obtained by EPTT to determine the virus titer in (TCID50/ml)

Virus	Virus titer before treatment	Virus titer after treatment	^a^Virus titer reduction	^b^Reduction factor
RSV	3.16 × 10^−3^	1.422 × 10^−2^	1 log	2.22 × 10
HSV-1	5.01 × 10^−3^	8.97 × 10^−1^	2 log	0.55 × 10^2^

Note: ^a^Virus titer reduction: the extract is considered active if the virus titer was reduced by 2 log. ^b^Reduction factor (RF): virus titer in the absence of drug over virus titer in the presence of drug. RF ≥ 10^3^ strong antiviral activity, RF = 10^2^ moderate antiviral activity and RF =10^1^ slight antiviral activity

### The virucidal, or Direct Virus Inactivation (DVI) and the prophylactic, or Inhibition of Virus Replication (IVR) by MBS extract

The neutralization and the inhibitory effects of MBS extract on virus replication (RSV and HSV-1) were studied. The study included incubation of the extract with virus specific cells (Vero and MRC-5) or with the virus itself. The results were obtained by microscopic examination for virus-induced CPE and the measurement of optical density by the MTT assay. The results of cytopathic reduction assay were expressed as mean of three independent experiments with five extract concentrations. As demonstrated in (Table 2), only the first concentration (220.96 mg/ml) of MBS extract inhibited the RSV-induced CPE completely at different incubation times while 25 % CPE started to develop at the concentration of 110.48 mg/ml when the virus was mixed with MBS extract and was then applied directly to Vero cells (zero hour time of incubation). The reduction of 50 % RSV-CPE was observed when the virus was incubated for zero hour with 55.24 mg/ml of MBS and for 1 h and 2 h with 27.62 mg/ml of MBS extract. On the other hand, when the extract was incubated for 30 min and 1 h with cells before virus infection 55.24 mg/ml of MBS extract produced 25 % of RSV-CPE in Vero cells whereas, 50 % CPE in Vero cells was observed after 30 min and 1 h with concentration 27.62 mg/ml of MBS extract (Table 2). The Lowest concentration of MBS extract enough to inhibit HSV-1 CPE completely after (zero, 1, and 2 h) of incubation with the virus before adding to MRC-5 cells was 136.58 mg/ml. In comparison to the direct effect of MBS extract on RSV, 25 % of HSV-1 CPE was observed with only 68.29 mg/ml of the extract when mixed together with the virus and applied directly to MRC-5 while MBS extract at concentrations 17.07 and 8.53 mg/ml were capable to inhibit 50 % CPE after 1 and 2 h of incubation with the virus. The indirect effect of MBS extract, which was estimated by incubating the extract with MRC-5 cells, was clearly different from the same effect on Vero cells. Here, the extract needed only the concentration of (17.07 mg/ml) to reduce the CPE to 50 % after 30 min and 1 h of incubation with the cells (Table 3).

**Table 2:** RSV-induced CPE (%) after incubating the MBS extract with the virus and with Vero cell line

Protocols	Time	^a^Conc.1	Conc.2	Conc.3	Conc.4	Conc.5
^b^DVI	0 h	0 %	0-25 %	25-50 %	50 %	75 %
	1 h	0 %	0 %	0-25 %	50 %	50-75 %
	2 h	0 %	0 %	0 %	50 %	75 %
^c^IVR	30 min	0 %	0 %	25-50 %	50 %	50-75 %
	1 h	0 %	0 %	0-25 %	25-50 %	50-75 %

Note: ^a^Conc.: extract concentration (220.96, 110.48, 55.24, 27.62, and 13.81 mg/ml). ^b^DVI: the direct virus inactivation. ^c^IVR: the inhibition in virus infection after specific cell treatment

**Table 3:** HSV-1 induced CPE (%) after incubating the MBS extract with the virus and with MRC-5 cell line

Protocols	Time	^a^Conc.1	Conc.2	Conc.3	Conc.4	Conc.5
^b^DVI	0 h	0 %	0-25 %	25-50 %	50-75 %	100 %
	1 h	0 %	0 %	0-25 %	25-50 %	50-75 %
	2 h	0 %	0 %	0 %	0-25 %	50 %
^c^IVR	30 min	0 %	0 %	25-50 %	50 %	≥75 %
	1 h	0 %	0 %	0-25 %	25-50 %	50-75 %

Note: ^a^Conc.: extract concentration (136.58, 68.29, 34.14, 17.07, and 8.53 mg/ml). ^b^DVI: the direct virus inactivation. ^c^IVR: the inhibition in virus infection after specific cell treatment

MBS extract at concentration of 220.96 mg/ml caused 50 % cytotoxicity (CC50) on Vero cells (specific cells for RSV) while only 136.58 mg/ml was enough to cause the 50 % death (CC50) in MRC-5 cell line (specific cells for HSV-1). The significant direct antiviral activity of MBS extract on RSV was observed when the extract incubated with the virus for 1 h then applied to Vero cells (IC50 = 15.62 mg/ml) in comparison to zero hour and 2 h of incubation which were found to be less active (*P* < 0.05). These significant findings were supported by the results of the SI, 14.18, for 1 h incubation time which was significantly higher than that of zero hour, 8.35, and 2 h, 8.28 (*P* < 0.05). These results supported our findings regarding the efficacy of 1 h incubation of virus with MBS. Regarding the incubation of MBS extract with Vero cells before the infection with RSV, the incubation time of one hour was also the proper time to inhibit 50 % of RSV-induced CPE (IC50 = 17.23 mg/ml) which gave SI of 12.82 when compared with 30 min of incubation. Therefore, 1 h time of incubation was found to be the proper time for both direct virucidal effect of MBS and indirect inhibition of virus replication by treating Vero cells with MBS. This incubation time was used to investigate the antiviral effect of the standard drug (Ribavirin) and compare it with MBS extract activity (Tables 4, 5 and 6). On the other hand, when incubating HSV-1 with MBS extract, it needed 2 h to inhibit 50 % of its CPE on MRC-5 cells (IC50 = 7.62 mg/ml) which was higher than that of zero or 1 h incubation times (*P* < 0.05). The SI, 18.23 for 2 h reflected the same result when compared to the SI of zero hour and 1 h, 5.69 and 9.91, respectively (*P* < 0.05). Two hours incubation was used to estimate the antiviral activity of the standard drug (Acyclovir) in order to be compared with that of MBS extract. MBS extract needed 1 h incubation time to induce its prophylactic effect against HSV-1 when incubated with MRC-5. It needed 1 h to give IC50 of 12.72 mg/ml and resulted in SI of 10.9. This effective time of incubation, *i.e.*, 1 h to provide the prophylactic effect of MBS extract was used to evaluate the same activity for Acyclovir. These results reflected the fact that the direct effect of MBS extract needed more time (about 2 h) to create effective SI against HSV-1 while it needed only 1 h to generate such effective SI against RSV (Tables 7, 8 and 9).

**Table 4:** The antiviral activity of MBS extract on RSV by the direct virucidal effect and the prophylactic effect on Vero cell line

Protocol	Time	^a^CC50 mg/ml	^b^IC50 mg/ml	^c^SI
^d^DVI	0 h	220.96 ± 60.56	26.98 ± 2.82	8.35 ± 0.79
	1 h	220.96 ± 60.56	15.62 ± 0.61	14.18 ± 0.54
	2 h	220.96 ± 60.56	26.72 ± 0.95	8.28 ± 0.29
Ribavirin	1 h	1.4 ± 0.088	0.06 ± 0.005	23.39 ± 2.44
^e^IVR	30 min	220.96 ± 60.56	18.92 ± 0.47	11.69 ± 0.28
	1 h	220.96 ± 60.56	17.23 ± 0.3	12.82 ± 0.23
Ribavirin	1 h	2.28 ± 0.057	0.087 ± 0.005	21.95 ± 2.12

Note: ^a^cytotoxic concentration 50, ^b^ Inhibitory concentration 50, ^c^ selective index ^d^DVI: the direct virus inactivation. ^e^IVR: the inhibition in virus infection after specific cell treatment

**Table 5:** The differences in 50 % inhibitory concentration (IC50) of MBS extract on RSV between the different times in the same protocol (DVI and IVR)

Protocol	Time vs Time	IC50 vs IC50	*P* value	
^a^DVI	0 h vs 1 h	26.98 ± 2.82 vs 15.62 ± 0.61	0.008	Significant
	0 h vs 2 h	26.98 ± 2.82 vs 26.72 ± 0.95	0.46	Non-Significant
	1 h vs 2 h	15.62 ± 0.61 vs 26.72 ± 0.95	0.0003	Significant
^b^IVR	30 min vs 1 h	18.92 ± 0.47 vs 17.23 ± 0.3	0.02	Significant

Note: ^a^DVI: the direct virus inactivation. ^b^IVR: the inhibition in virus infection after specific cell treatment

**Table 6:** The differences in selectivity index (SI) of MBS extract on RSV between the different times in the same protocol (DVI and IVR)

Protocol	Time vs Time	SI vs SI	*P* value	
^a^DVI	0 h vs 1 h	8.35 ± 0.79 vs 14.18 ± 0.54	0.001	Significant
	0 h vs 2 h	8.35 ± 0.79 vs 8.28 ± 0.29	0.47	Non-Significant
	1 h vs 2 h	14.18 ± 0.54 vs 8.28 ± 0.29	0.0003	Significant
^b^IVR	30 min vs 1 h	11.69 ± 0.28 vs 12.82 ± 0.23	0.018	Significant

Note: ^a^DVI: the direct virus inactivation. ^b^IVR: the inhibition in virus infection after specific cell treatment

**Table 7:** The antiviral activity of MBS extract on HSV-1 by the direct virucidal effect and the prophylactic effect on MRC-5 cell line

Protocol	Time	^a^CC50 mg/ml	^b^ IC50 mg/ml	^c^SI
^d^DVI	0 h	136.58 ± 55.08	24.17 ± 1.43	5.69 ± 0.35
	1 h	136.58 ± 55.08	13.8 ± 0.44	9.91 ± 0.31
	2 h	136.58 ± 55.08	7.62 ± 0.71	18.23 ± 1.69
Acyclovir	2 h	0.35 ± 0.005	0.014 ± 0.0005	22.56 ± 1.11
^e^IVR	30 min	136.58 ± 55.08	15.53 ± 0.74	8.83 ± 0.42
	1 h	136.58 ± 55.08	12.72 ± 1.12	10.9 ± 0.96
Acyclovir	1 h	0.37 ± 0.005	0.02 ± 0.003	15.04 ± 1.9

Note: a cytotoxic concentration 50, b Inhibitory concentration 50, c selective index ^d^DVI: the direct virus inactivation. ^e^IVR: the inhibition in virus infection after specific cell treatment

**Table 8:** The differences in the 50 % inhibitory concentration (IC50) of MBS extract on HSV-1 between the different times in the same protocol (DVI and IVR)

Protocol	Time vs Time	IC50 vs IC50	*P* value	
^a^DVI	0 h vs 1 h	24.17 ± 1.43 vs 13.8 ± 0.44	0.001	Significant
	0 h vs 2 h	24.17 ± 1.43 vs 7.62 ± 0.71	0.0002	Significant
	1 h vs 2 h	13.8 ± 0.44 vs 7.62 ± 0.71	0.0009	Significant
^b^IVR	30 min vs 1 h	15.53 ± 0.74 vs 12.72 ± 1.12	0.05	Non-Significant

Note: ^a^DVI: the direct virus inactivation. ^b^IVR: the inhibition in virus infection after specific cell treatment

**Table 9:** The differences in selectivity index (SI) of MBS extract on HSV-1 between the different times in the same protocol (DVI and IVR)

Protocol	Time vs Time	SI vs SI	*P* value	
^a^DVI	0 h vs 1 h	5.69 ± 0.35 vs 9.91 ± 0.31	0.0004	Significant
	0 h vs 2 h	5.69 ± 0.35 vs 18.23 ± 1.69	0.0009	Significant
	1 h vs 2 h	9.91 ± 0.31 vs 18.23 ± 1.69	0.004	Significant
^b^IVR	30 min vs 1 h	8.83 ± 0.42 vs 10.9 ± 0.96	0.06	Non-Significant

Note: ^a^DVI: the direct virus inactivation. ^b^IVR: the inhibition in virus infection after specific cell treatment

### Comparison between the virucidal and the prophylactic properties of MBS extract to RSV, and between that of MBS extract and Ribavirin

The proper incubation time of each protocol for MBS extract is compared together to find the best antiviral activity. MBS extract was effective as both prophylactic and virucidal agent; however, it was a bit better as a virucidal agent on RSV with (IC50 = 15.62 mg/ml) than as prophylactic agent. This was shown by SI values which were 14.18 and 12.82 for virucidal and prophylactic activities, respectively (Tables 10 and 11).

**Table 10:** Comparisons of the 50 % inhibitory concentration (IC50) of MBS extract on RSV at the best time for each protocol

Protocol vs Protocol	Time vs Time	IC50 vs IC50 mg/ml	*P* value	
^a^IVR vs ^b^DVI	1 h vs 1 h	17.23 ± 0.3 vs 15.62 ± 0.61	0.03	Significant

Note: ^a^IVR: the inhibition in virus infection after specific cell treatment. ^b^DVI: the direct virus inactivation

**Table 11:** Comparisons of the selectivity index (SI) of antiviral activity on RSV between prophylactic (IVR) and virucidal (DVI) at best time for MBS extract and between that of MBS extract and the standard drug (Ribavirin)

Extract	Protocol vs Protocol	Time vs Time	SI vs SI	*P* value	
MBS	^a^IVR vs ^b^DVI	1 h vs 1 h	12.82 ± 0.23 vs 14.18 ± 0.54	0.04	Significant
MBS vs Ribavirin	^a^IVR vs ^a^IVR	1 h vs 1 h	12.82 ± 0.23 vs 21.95 ± 2.12	0.006	Significant
MBS vs Ribavirin	^b^DVI vs ^b^DVI	1 h vs 1 h	14.18 ± 0.54 vs 23.39 ± 2.44	0.01	Significant

Note: ^a^IVR: the inhibition in virus infection after specific cell treatment. ^b^DVI: the direct virus inactivation

Comparing with the anti-RSV activity of Ribavirin, there were significant differences in the prophylactic and virucidal effects of MBS extract and Ribavirin (*P* < 0.05) at the same time of incubation. The results revealed that the SI of MBS extract as prophylactic agent (SI = 12.82) was less than that of Ribavirin (SI = 21.95). Furthermore, the level of MBS extract as a virucidal agent (SI = 14.18) was lower than that of Ribavirin (SI = 23.39), (Table 11).

### Comparison between the virucidal and the prophylactic properties of MBS extract to HSV-1, and between that of MBS extract and Acyclovir

By using the best incubation time that generated a significant antiviral activity against HSV-1, the prophylactic and virucidal effects of MBS extract were compared together. At time of incubation (2 h), MBS extract was effective as virucidal and prophylactic agent but it was most prominent as a virucidal agent against HSV-1 using 7.62 mg/ml MBS extract (Table 12). As shown in (Table 12), there were significant differences (*P* < 0.05) when comparing the SI values between different protocols for MBS extract. In other words, the SI value, 18.23, of the virucidal activity of MBS extract was significantly higher than that of the prophylactic action.

**Table 12:** Comparisons of the 50 % inhibitory concentration (IC50) of MBS extract on HSV-1 at the best time for each protocol

Protocol vs Protocol	Time vs Time	IC50 vs IC50	*P* value	
^a^IVR vs ^b^DVI	1 h vs 2 h	12.72 ± 1.12 vs 7.62 ± 0.71	0.009	Significant

Note: ^a^IVR: the inhibition in virus infection after specific cell treatment. ^b^DVI: the direct virus inactivation

In addition, MBS extract did not show any significant difference when its prophylactic and virucidal activities were compared with that of Acyclovir (*P* > 0.05), (Table 13).

**Table 13:** Comparisons of the selectivity index (SI) of antiviral activity on HSV-1 between prophylactic (IVR) and virucidal (DVI) at best time for MBS extract and between that of MBS extract and the standard drug (Acyclovir)

Extract	Protocol vs Protocol	Time vs Time	SI vs SI	*P* value	
MBS	^a^IVR vs ^b^DVI	1 h vs 2 h	10.9 ± 0.96 vs 18.23 ± 1.69	0.009	Significant
MBS vs Acyclovir	^a^IVR vs ^a^IVR	1 h vs 1 h	10.9 ± 0.96 vs 15.04 ± 1.9	0.06	Non-significant
MBS vs Acyclovir	^b^DVI vs ^b^DVI	2 h vs 2 h	18.23 ± 1.69 vs 22.56 ± 1.11	0.05	Non- Significant

Note: ^a^IVR: the inhibition in virus infection after specific cell treatment. ^b^DVI: the direct virus inactivation

### The antiviral cytokines produced by the treated-viral infected cells

**Fig. 1:**
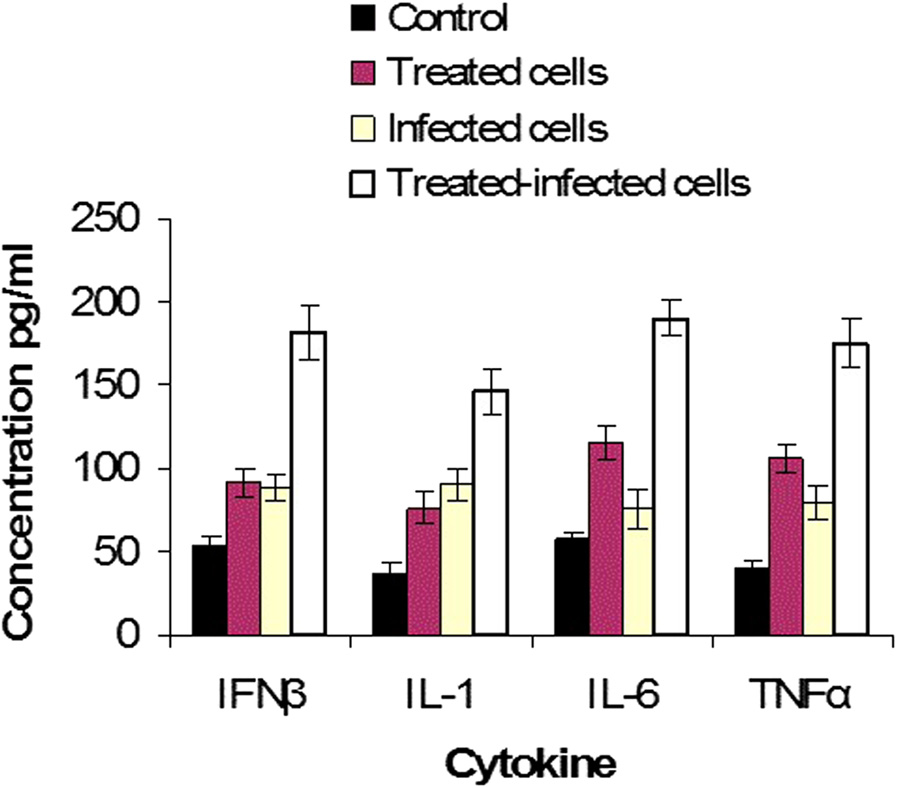
A histogram shows the mean ± 2SE of the extracellular concentration of IFNβ, IL-1, IL-6, and TNFα cytokines in the supernatant of cultured control, MBS extract treated, HSV-1 infected, and MBS extract treated & HSV-1 infected MRC-5 cells

The extracellular concentration of a group of cytokines implicated in the antiviral response was monitored in the supernatant of MRC-5 cells. The cells were categorized into four groups. The first group was MRC-5 cells cultured without exposure to either extract or viral infection by HSV-1. The second group was MRC-5 cells treated for one hour with IC50 of IVR protocol of MBS extract alone. The third group was MRC-5 cells infected with HSV-1. The fourth group was MRC-5 cells pretreated with IC50 of IVR protocol of MBS extract and cells were then infected with HSV-1. Both the group of MBS treated cells and the group of infected cells showed higher synthesis of all tested cytokines, IFNβ, IL-1, IL-6, and TNFα than in control cells (MBS treated cells: *P* = 0.005, 0.011, <0.001, <0.001; infected cells: *P* = 0.009, 0.006, 0.041, 0.01, respectively). Moreover, the level of IFNβ and IL-1 was not significantly different between MBS treated cells and infected cells (*P* = 0.75, 0.31, respectively) while the level of IL-6 and TNFα was higher in MBS treated cells than in infected cells (*P* = 0.009, 0.025, respectively). These results indicated that MBS extract can induce the synthesis of IL-6 and TNFα more strongly than the synthesis of IFNβ and IL-1 (*P* < 0.05), (Table 14 and Fig. 1). Above all, MBS extract induced synthesis of all tested cytokines in MRC-5 cells infected with HSV-1 far higher than all other groups (*P* < 0.001), (Table 14 and Fig. 1). Moreover, MBS extract showed a powerful prophylactic effect for MRC-5 cells against viral infection by charging the MBS treated cells to be ready and highly responsive against any viral infection.

**Table 14:** The measured concentrations of antiviral cytokines in control, MBS extract treated, HSV-1 infected, and MBS extract treated & HSV-1 infected MRC-5 cells

Cytokine	Control cells Mean ± 2SE (pg/ml)	Treated cells Mean ± 2SE (pg/ml)	Infected cells Mean ± 2SE (pg/ml)	Treated-infected cells Mean ± 2SE (pg/ml)
IFNβ	53.7 ± 5.72	91.78 ± 7.98	88.49 ± 7.49	181.42 ± 15.6
IL-1	36.84 ± 6.22	76.16 ± 9.24	90.58 ± 9.32	145.72 ± 13.51
IL-6	58.11 ± 3.85	115.24 ± 10.1	75.92 ± 11.54	190.31 ± 10.6
TNFα	40.3 ± 4.81	105.97 ± 8.42	79.43 ± 10.04	174.67 ± 14.77

## Discussion

Flavonoids, the most common polyphenols found in plants, have been associated with biological effects such as antibacterial, antiviral, anti-inflammatory, antiplatelet, antioxidant, free radical scavenging, and vasodilatory effects [9, 10]. MBS is known to have high antioxidant activity which may be correlated with an effective antiviral activity.

To establish the efficacy of the antiviral activity of MBS extract, virus yield reduction assay was performed in which plant extract was added to host cells (Vero and MRC-5) after infection with RSV and HSV-1, respectively. The maximum non-cytotoxic concentration of MBS extract was able to reduce HSV-1 by 2 log (RF = 0.55 × 10^2^) and RSV only by 1 log (RF = 2.22 × 10) to give a moderate and slight antiviral activity against HSV-1 and RSV, respectively. These results reflected the efficacy of the solvent, *i.e.*, methanol, in extracting the antiviral components from MBS. Moreover, the synergistic effect of these extracted polyphenols may play a critical role in this activity. It was reported that methanol is the best solvent for the consistent extraction of antimicrobial substances from medicinal plants compared to other solvents such as water, ethanol, or hexane [11]. The benefit of crude extract over the fractionated extract is that the crude one is composed of a large mixture of polyphenols that could interplay effectively giving rise to novel and antiviral activities. It was stated that different polyphenolic components of a plant extract may influence different steps of antimicrobial activity in a synergistic manner [12].

MBS extract reduced virus titer in one to two logs. Plant extracts are considered active if the virus titer was reduced by one log and highly active by two logs after treatment [7, 13]. Furthermore, any extract with RF value of ≥ 10 should be considered highly valuable and selected to determine the mode of its antiviral action [14]. For these reasons, MBS extract was further analyzed to investigate its antiviral activities as well as their modes of action to reduce RSV and HSV-1 titer.

The results showed that the antiviral activity of MBS extract is dose-dependent. Concentrations of extract close to the CC50 caused 100 % inhibition of CPE. However, much lower concentrations succeeded in reducing virus CPE in large extent giving clue on the potent antiviral activity in MBS extract. MBS extract at concentration of 220.96 mg/ml caused death to 50 % of Vero cells and at 136.58 mg/ml caused death in 50 % of MRC-5 cells. Therefore, CC50 values of MBS extract on both Vero and MRC-5 cells are considered too high when compared to the concentrations yielded 50 % antiviral activity, IC50, which ranged from 7.62 to 15.62. These figures of IC50 indicated high selectivity in the extract activity. To elucidate this aspect, it was necessary to calculate the selectivity index, *i.e.*, the therapeutic index. It is generally considered that biological efficacy is not due to *in vitro* cytotoxicity when SI ≥ 10 [15, 16].

The results of MBS extract cytotoxicity on the virus host cells (Vero and MRC-5 cells) were in correlation with the effective concentration which was needed to inhibit virus-induced CPE. The study’s results found that MBS extract was needed in lower concentrations to inhibit HSV-1 induced CPE than that needed to inhibit RSV-induced CPE. This depended on the CC50 values of MBS extract on MRC-5 and Vero cells. The results disclosed the fact that MBS extract was more cytotoxic to MRC-5 cells (CC50 = 136.58 mg/ml) than to Vero cells (CC50 = 220.96 mg/ml). In other words, Vero cells can tolerate higher MBS extract concentrations than can MRC-5 cells. Fortunately, the high cytotoxicity of MBS extract against MRC-5 was accompanied with high antiviral activity against HSV-1 leading to attain low working antiviral concentrations much lower than the cytotoxic concentrations for the host cells. The maximum non-cytotoxic concentrations (CC50) of MBS extract for both Vero and MRC-5 cells showed significant reduction of RSV- and HSV-1- induced CPE by 100 %. This can be attributed to the cytotoxicity of the extract used for the host cells; however, the lower 2-fold concentration of the MBS extract showed the same 100 % inhibition of viral CPE for treatments 1 h and 2 h. This indicated a specific antiviral activity rather than viral reduction due to cytotoxicity of host cells.

The IVR treatments by MBS extract showed optimal time of 1 h rather than 30 min for both Vero and MRC-5 cells while in DVI treatments, 1 h and 2 h were optimal for RSV and HSV-1, respectively. Accordingly, 2 h were enough for HSV-1 while just 1 h was enough for RSV. This provided evidence that HSV-1 needs longer exposures than RSV with antiviral agents to respond efficiently.

The SI of MBS extract after 1 h of incubation was quite high (14.18), pointing out to a high selectivity in the extract action. Accordingly, 1 h of RSV treatment with MBS extract was the proper time to inhibit virus-induced CPE by 50 % with much lower cytotoxicity on the host cells (Vero cells) and significant selectivity on the virus. In addition, the SI of MBS extract treatment for Vero cells before being infected with RSV, namely, IVR protocol, was (12.82), which indicated also a high selectivity in the extract action over the cytotoxicity to Vero cells. The activity of MBS extract on RSV in this study may agree with a previous study which found that the methanol crude extract of some plants is highly effective to inhibit RSV and can be considered the best choice in searching for novel antiviral agents for RSV [17].

The selectivity, SI, of 2 h treatment of MBS extract for HSV-1 was very high (18.23) while SI for 1 h treatment, *via* IVR, of MBS extract to the host cells (MRC-5 cells) before being infected with HSV-1 was just acceptable, 10.9. The antiviral activities of MBS extract in both DVI and IVR for RSV in Vero cells and HSV-1 in MRC-5 cells gave many indications. First, MBS extract acted as a potent antiviral agent and its action was not related to the cytotoxicity of host cells. Second, MBS extract was highly selective against RSV and HSV-1 or against the virally infected cells. Third, MBS extract, acted as virucidal agent more than prophylactic agent. These valuable results of MBS extract anti-HSV1 activity may be supported by a recent study on plant methanol crude extract which found that such extract found to be effective to inhibit HSV-1 infection *in vitro* [18]. The results of MBS extract, IC50 and SI, with different modes of action against RSV were significantly better in its virucidal action (IC50 = 15.62 and SI = 14.18) than its prophylactic action (IC50 = 17.23 and SI = 12.82). Similarly, MBS extract was a virucidal agent (IC50 = 7.62 and SI = 18.23) rather than a prophylactic agent (IC50 = 12.72 and SI = 10.9) against HSV-1. These findings may point out to the importance of MBS extract as an agent with the ability to interact with viral envelope more sufficiently than its ability to interact with host cell surface. One of the clear reasons that may explain the effective antiviral activity of MBS extract in the current study is the presence of high level of antioxidant compounds in the germinated mung bean sprout. Germination of the mung bean causes a rise in total content of the antioxidant components like phenolic compounds, α-tocopherol and vitamin C [19, 20]. It is well known that α-tocopherol, a member of vitamin E family, has a potent antioxidant activity [21, 22]. Moreover, many studies proved the efficacy of α-tocopherol as antithrombotic, anticoagulant, neuroprotective, antiproliferative, immunomodulatory, cell membrane-stabilizing and antiviral [23–25]. On the other hand, ascorbate (Vitamin C) has been shown to have specific antiviral effect in which it inactivates RNA or DNA of viruses [26–28]. Moreover, ascorbate was found to be able to act synergistically with other phenolic compounds to enhance their antiviral activity [29]. All these facts may explain the effective antiviral activity of MBS extract. The ability of each antioxidant compound to work alone or with other compounds to enhance its effect in the antiviral activity was well proven by some studies [23, 24, 26]. These studies suggested that the potent antioxidant activity that leads to antimicrobial activity is a result of a combination of different compounds having synergistic effect [30].

The acute respiratory illness due to RSV is one of the most common causes of hospitalization in very young children worldwide and treatment remains supportive. Ribavirin is approved for the treatment of severe RSV disease; however, its effectiveness in improving outcomes is questionable [31–33]. Additionally, the clinical treatment with Ribavirin requires high doses with significant side effects including thrombocytosis and severe anemia [34, 35]. All these factors together raise the critical need for a new, safe, and effective antiviral agent to win the battle against RSV.

Despite the fact that MBS extract worked as a virucidal agent (SI = 14.18) more powerfully than as a prophylactic agent (SI = 12.82) against RSV, this activity was not better than the activity of Ribavirin. Both DVI and IVR antiviral activities of MBS extract showed significant differences from that of Ribavirin. Thus, MBS extract had effective virucidal activity against RSV (SI = 14.18) but it is still not much like the virucidal activity of Ribavirin (SI = 23.39). However, MBS extract might harbor some advantages over ribavarin such as safety, low cost, and its natural origin which together allow using MBS extract, and/or the underlying antiviral component, in higher doses and longer periods than ribavarin; this ultimately leads to equal or superior therapeutic values of MBS extract to that of ribavarin. For these reasons, the current trend of searching antimicrobial has moved to the natural products for the direct use of natural extracts or the use of the responsible antimicrobial component.

HSV-1 can establish latent infection in the nervous system and usually leads to life-threatening diseases in immunocompromised individuals upon reactivation [36]. Treatment with conventional nucleoside analogues such as Acyclovir is effective in most cases, but drug-resistance may arise due to prolonged treatment in immunocompromised individuals [37]. The recovery of the same Acyclovir-resistant virus during recurrent infections suggests that Acyclovir-resistant HSV-1 establishes latency and reactivates intermittently in highly risk individuals [38]. Moreover, a well-known side effect of prolonged Acyclovir treatment is kidney nephrotoxicity [39]. Accordingly, it is conceived so far that the plants’ derived anti-HSV1 drugs might be the only way that can solve these problems.

MBS extract showed higher virucidal than prophylactic activities against HSV-1. However, both activities were well evident. The virucidal activity against HSV-1 was high (SI = 18.23) and the prophylactic activity was fair enough to be considered effective (SI = 10.9) against HSV-1 infection. But the most interesting, there was no significant difference found between the prophylactic and the virucidal actions of MBS extract and that of the HSV-1 standard drug, Acyclovir. Accordingly, MBS extract could be used in the treatment and/or in the prophylaxis of HSV-1 against HSV-1 infection at comparable levels to that of Acyclovir.

MBS extract showed capability for killing HSV-1 and RSV viruses which is a sign for a prominent virucidal activity. In addition, it exerted antiviral prophylactic activity in both Vero cells and MRC-5 host cells against both RSV and HSV-1 viruses, respectively. Therefore, there was a need to elucidate part of the underlying mechanism(s) exerted by this extract on protecting virus-specific host cells against the infection and cytopathic effects of the tested viruses. For this goal, the induction for the synthesis of four cytokines known as actively engaged in the antiviral activity of mammalian cells against RNA and DNA viruses, namely IFNβ, IL-1, IL-6, and TNFα, was studied [40–47].

Starting with the most prominent antiviral cytokine, IFNβ, this cytokine was remarkably induced by MBS extract in extract-pretreated HSV-1-infected MRC-5 cells. In addition, this extract induced IFNβ expression in non-infected cells treated with this extract. These findings provided evidence on the ability of MBS extract to charge human cells ready for any concurrent and subsequent viral infections. However, the interesting feature which was found here that MBS extract induced IFNβ in non-infected cells much lower than in infected cells. This feature adds privilege on using MBS extract or the antiviral components that will be isolated from it because these agents selectively induce IFNβ most highly in infected cells only. Therefore, using MBS extract might override the side effects of using interferons therapy which usually causes febrile reaction, headache, and unwanted systematic immune reactions [48, 49].

For TNFα, this cytokine was recently found to be important antiviral as well as anticancer cytokine [41, 43, 50–52]. MBS extract showed good inducing capacity for TNFα expression by MRC-5 cells. The highest level of TNFα induction by MBS extract was observed in treated-infected cells followed by treated cells, infected cells, and finally by control cells (non-treated non-infected cells). These findings granted clues on the ability of MBS extract to selectively induce higher levels of TNFα by human cells when these cells are being infected by viruses. Therefore, MRC-5 cells needed two signals for the expression of the maximum levels of TNFα; these signals came from the extracts’ pretreatment and HSV-1 infection. Like the case of IFNβ, the relatively selective induction of TNFα expression in virally infected human cells pretreated with MBS extract can also override the systemic side effects for using synthetic TNFα products [53]. Wei *et al.* found that TNFα levels, locally and systematically, increase significantly in mice with HSV-1 infection; moreover, after treatment with Acyclovir for HSV-1 infection, the level of TNFα was largely decreased [54]. This report revealed the pivotal role of TNFα in counteracting DNA viruses such as HSV-1. As a proof for the strong link between TNFα induction and viral infection, mainly by HSV-1, a previous study [43] found that TNFα and IL-1 cytokines were produced largely by microglial cells during non-productive infection while other cytokines, including interferons, were not induced by such weak infective cycle. It was revealed that TNF induce multiple antiviral mechanisms, and to synergize with interferon (IFN)-gamma in promoting antiviral activities; and the antiviral activity of TNF is mediated *via* both TNF receptors, TNFR1 (p55) and TNFR2 (p75), *in vivo* [55]. Recently, it was found that the combination of TNFα and IFNβ induces a novel synergistic antiviral state that is highly distinct from that induced by either cytokine alone [56]. Hence, MBS extract is believed to exert a synergistic antiviral effect by inducing both TNFα and IFNβ cytokines.

Several reports proved the antiviral role of IL-1 and IL-6 in human and murine cells [43, 46, 47, 57–60]. The expression of IL-1 was highest in treated-infected cells, followed equally by treated cells alone and infected cells alone, and finally the control cells. For IL-6, the highest expression was in treated-infected cells, followed by treated cells, infected cells, and control cells. Like the case of TNFα and IFNβ, MBS extract induced the highest level of expression of IL-1 and IL-6 in HSV-1 infected cells already treated with the extract. Thus MBS extract showed selectivity in inducing all tested cytokines. This phenomenon points out to the possibility that TNFα, IFNβ, IL-1, and IL-6 are induced by MBS extract *via* the same mechanism(s). One of the proposed mechanisms to induce all these cytokines to create antiviral resistance is the induction of nuclear factor-kappa B (NF-kappa-B), transcriptional factor. Recent reports stated and proved the antiviral role of NF-kappa-B [61, 62]. Moreover, antiviral resistance against HSV-1 *via* TNFα was found to be attributed to NF-kappa-B [42].

## Conclusion

The primary antiviral assay revealed noticeable antiviral activities against RSV and HSV-1 by MBS extract. The SI reflected high selectivity and low cytotoxicity of MBS extract over RSV, Vero cells, HSV-1, and MRC-5, respectively. MBS extract was more effective as a virucidal agent than a prophylactic agent on RSV and HSV-1. Hence, MBS extract can be used in the treatment and in the prophylaxis against both HSV-1 and RSV infection with comparable levels to that of standard drugs but with much more safety. MBS extract revealed ability to create a highly inducible status in virally infected cells for the expression of high levels of IFNβ, TNFα, IL-1, and IL-6 cytokines which may explain in part its prophylactic activity. These cytokines proved to play essential and synergistic role in the antiviral resistance of human cells. Collectively, the importance of these results comes from their novelty. The effective anti-RSV and anti-HSV1 activities of MBS extract were significant. The findings of this study may open the gate wide to develop new antiviral agents from inexpensive and safe sources like mung bean sprout.

## Methods

### Preparation of the MBS extract

Fresh mung bean sprouts (MBS), devoid of any preservative antimicrobials, was purchased from local markets and were identified by the leader of the research team, namely Dr. Fatimah Abu Bakar. A voucher specimen of MBS has not been deposited in a publicly available herbarium as MBS used is that of the daily used produce. The mung bean sprouts were left to dry in dark area for three days at room temperature 23-25 °C. After the dryness of sprouts, they were ground to powder. The ground powder was extracted 1:10 wt/v with 2.4 M HCl acidified methanol (Merck, Darmstadt, Germany) to extract all components of phenolic compounds, free and conjugated, [63]; the ground powder was then soaked in dark area for three days at room temperature. The supernatant was collected after filtration and the fresh solvent was added to the plant material. The extraction procedure was repeated twice and the collected extract was evaporated to dryness under vacuum at 40 °C by using rotary evaporator. To remove the effect of the acidity from the crude extract, the crude extract was neutralized. The pH of MBS extract was adjusted to be neutral ranging from 6.8 to 7.0. The dried extract was stored at −18 °C in a desiccant form until further use.

### Preparation of the stock extract

The stock extract for MBS was prepared by redissolving in dimethyl sulfoxide (DMSO) 0.1 %, (BIO BASIC INC., NY, USA). This concentration was shown to be non toxic to cell culture [64]. The dissolved suspension was centrifuged at 134 g for 10 min and filtrated by 0.22 μm Millipore filters (Nalgene, UK). The stock was stored at −20 °C until it further use. The concentration of the stock extract was determined as required.

### Specific cells propagation and cryopreservation

African green monkey kidney cells (Vero; ATCC CCL-81) were used to culture human respiratory syncytial virus (RSV strain A-2: ATCC VR-1540) and human herpes simplex virus type 1 (HSV-1 strain HF: ATCC VR-260) while human embryonic lung fibroblast cells (MRC-5; ATCC CCL-171) were used to culture human herpes simplex virus type 1 (HSV-1 strain HF: ATCC VR-260) only. Cells were propagated as monolayer at 37 °C under a humidified 5 % CO_2_ atmosphere in Roswell Park Memorial Institute-1640 (RPMI-1640) culture medium w/L-glutamine (biowest, Florida, USA) supplemented with Fetal Bovine Serum (FBS) 10 %, (Sigma, Germany), 50 U/ml penicillin-streptomycin (biowest, Florida, USA), and 2.5 μg/ml amphotericin B (biowest, Florida, USA). Part of the cells were cryopreserved for future work in liquid nitrogen (−196 °C) after suspending them in RPMI-1640 cryospreserved medium supplemented with FBS 10 %, DMSO 20 %, 50 U/ml of penicillin-streptomycin, and 2.5 μg/ml amphotericin B.

### Virus propagation (RSV and HSV-1)

RSV and HSV-1 were propagated on 70-80 % and 90 % confluent Vero cell monolayer, respectively, in RPMI-1640 maintenance medium with 2 % FBS and antibiotics as described above. Viruses were stored at −80 °C until they were used again.

### RSV titration by endpoint titration technique

Vero cells of 1 × 10^5^ cell/well were grown in RPMI-1640 culture medium in 96-well flat-bottom tissue culture plates (Orange Scientific, Europe). After 24 h of incubation, the medium was removed and the monolayer cells were incubated for one and half hour at 37 °C with serial 10-fold diluted virus suspensions in phosphate buffer saline (PBS), (CALBIOCHEM, Darmstadt, Germany). After adsorption, the inoculum was removed and 200 μl/well RPMI-1640 maintenance medium with 2 % FBS were added. Negative Control wells represented Vero cells without virus. All plates were incubated at 37 °C in a humidified 5 % CO_2_ atmosphere and were observed daily for cytopathic effect (CPE). Estimation of the endpoints was made after 48 h. Titers were calculated as 50 % tissue culture infectious doses (TCID50)/ml, using the Reed and Muench method [65] to estimate endpoints. The titration was repeated for three times with four replicates for each virus dilution at each time.

### HSV-1 titration by plaque assay

HSV-1 was titrated using plaque assay. For this purpose Vero cells of 1 × 10^5^ cell/well were grown in RPMI-1640 culture medium using 6-well tissue culture plates (Orange Scientific, Europe). After 24 h, the medium was removed and the monolayer cells were incubated with serial 10-fold diluted virus suspensions in PBS for 1 h at 37 °C. After removing the inoculum, the infected and non infected cells were overlaid with RPMI-1640 culture medium containing 0.5 % agarose type VII (Sigma, Steinheim, Germany). Negative control wells represented Vero cells without virus. After 48 h, the infected cells were fixed by 1 % formaldehyede (Sigma, Steinheim, Germany) for three days at room temperature. Afterwards, the agarose layer was removed and the infected cells were stained by 0.05 % neutral red (Sigma, Steinheim, Germany) for 2 h at room temperature. Plaques were counted and virus titers expressed as plaque forming units (PFU)/ml [36, 66]. The titration was repeated for three times with two replicates for each virus dilution at each time.

### Cytotoxicity assay

The cytotoxic activities of MBS extract and standard antiviral drugs on virus specific cells were evaluated using MTT assay. In this colorimetric assay, yellow MTT 3-[4,5-dimethylthiazol-2-yl]-2,-diphenyltetrazolium bromide, (CALBIOCHEM, Darmstadt, Germany) is reduced to purple formazan by live cells’ mitochondrial enzymes [67, 68] and the color change can be quantified by measuring the absorbance at certain wavelength. After incubating the virus-specific cells with each extract or the standard drugs, 50 μl MTT (5 mg/ml) and 200 μl of RPMI-1640 culture medium were added to each well. The plates were incubated in a humidified 5 % CO_2_ atmosphere for 4 h at 37 °C. Later, 200 μl/well DMSO was added to solublize the formazan crystal. The plates were shaked for 10 min and the absorbance was measured at 540 nm (reference 690 nm) using a 96-well plate ELISA reader (Sunrise Basic Tecan, Grödig, Austria). Each experiment was repeated for three times with four wells per dilution in each run. The 50 % cytotoxic concentration (CC50) was defined as the highest concentration that caused 50 % death or was cytotoxic to 50 % of virus-specific cells. Cytotoxicity was calculated using the following formula:

$$ \mathrm{Cytotoxic}\ \% = 100\ \hbox{--}\ \left[\left(\mathrm{O}\mathrm{D}\mathrm{t}\ /\ \mathrm{O}\mathrm{D}\mathrm{c}\right) \times 100\right] $$

Where (ODt) indicates the optical density of the treated cells and (ODc) indicates the optical density of the negative control or the untreated cells [15].

### The cytotoxic effects of MBS extract on vero and MRC-5 cells

Vero cells were cultivated as 1 × 10^5^ cell/well in 96-well flat bottom tissue culture plates. MRC-5 cells were cultivated as 3 × 10^4^ cell/well in 96-well flat bottom tissue culture plates. After 24 h of incubation at 37 °C in a humidified 5 % CO_2_ atmosphere, the monolayer cells were exposed to 2-fold serial dilutions of MBS extract in RPMI-1640 maintenance medium. Stock extract (600 mg/ml) was used in this assay. MBS serial dilutions ranged from 300 to 9.37 mg/ml. Each well contained 100 μl of extract dilution and 100 μl of RPMI-1640 maintenance medium. The negative control wells contained 100 μl of solvent (0.1 % DMSO) and 100 μl of RPMI-1640 maintenance medium. All the plates were incubated for another 24 h in the same conditions. Afterwards, the wells’ contents were removed and replaced with 200 μl/well maintenance medium; then, plates were incubated for 48 h at the same conditions. The effects of the extract were monitored daily though microscopic examination.

### The cytotoxic effect of the standard drugs ribavirin and acyclovir

The cytotoxic activity of the RSV standard drug Ribavirin (Sigma, Steinheim, Germany) and the cytotoxic effect of the HSV-1 standard drug Acyclovir (Sigma, Steinheim, Germany) were determined in order to calculate the CC50 for these two standard drugs. Vero (1 × 10^5^ cell/well) and MRC-5 (3 × 10^4^ cell/well) cells were cultured in 96-well flat bottom tissue culture plates with RPMI-1640 culture medium. After 24 h of incubation at 37 °C in humidified 5 % CO_2_, all the wells’ contents were removed and the cells were incubated with 2-fold serial dilutions of Ribavirin and Acyclovir prepared in RPMI-1640 maintenance medium with final volume of 200 μl/well. Negative control wells contained cells with maintenance medium (200 μl/well). All the plates were incubated for 24 h at the same conditions. The Ribavirin and Acyclovir stock solutions (1 mg/ml and 0.06 mg/ml, respectively) were prepared in PBS solution at serial dilutions ranged from 0.5 to 0.007 and 0.03 to 0.0005 mg/ml, respecitvely. After 24 h, the wells’ contents were removed and replaced with 200 μl/well maintenance medium. The plates were incubated for another 48 h at the same conditions. The cells condition was microscopically checked daily.

### Virus yield reduction assay

The antiviral activities of MBS extract on RSV and HSV-1 yields were evaluated to calculate the virus titer reduction factor (RF). RF represents the virus titer in the absence of a drug over the virus titer in the presence of a drug. RF ≥ 10^3^ shows a strong antiviral activity, RF = 10^2^ shows a moderate antiviral activity and RF =10^1^ shows slight antiviral activity [13, 14].

### RSV and HSV-1 yield reduction assays

This experiment was performed according to the method described by [69] with some modifications for RSV and by Suzutani and Behbahani and with some modifications [70, 71] for HSV-1. For RSV, Vero cells were cultivated as 1 × 10^5^ cell/well in 96-well flat bottom culture plates in RPMI-1640 culture medium and for HSV-1, MRC-5 cells were cultivated as (3 × 10^4^ cell/well) in 96-well flat bottom culture plates in RPMI-1640 culture medium. After 24 h of incubation at 37 °C in a humidified 5 % CO_2_ atmosphere, the medium was removed and the cell monolayer was exposed to 100 μl/well of RSV virus suspension in PBS of 3.16 × 10^3^ TCID50/ml for 1.30 h and in the case of HSV-1, the virus suspension was in PBS as 5.01 × 10^3^ PFU/ml for 1 h.

The virus suspension was removed and replaced with 200 μl/well of the extract non-cytotoxic 2-fold serial dilutions (100 μl/well) and RPMI-1640 maintenance medium (100 μl/well). Only 200 μl/well of RPMI-1640 maintenance medium was added to the positive and negative control wells. The positive control wells contained cells with virus without treatment. The negative control wells contained cells with maintenance medium only without virus and without treatment. For RSV, the stock extract of MBS used was 441.92 mg/ml at serial dilutions ranging from 220.96 to 13.81 mg/ml while, for HSV-1, the stock solution was 273.16 mg/ml at serial dilutions ranging from 136.58 to 8.53 mg/ml. All the plates were incubated in a humidified 5 % CO_2_ atmosphere for 48 h (RSV) and 24 h (HSV-1) at 37 °C. Later, three steps of freezing at −80 °C and thawing at 37 °C were performed (10 min for each step). The cell monolayer was disrupted and transferred to 1.5 microtubes (Eppendrof, Hamberg, Germany); then it was centrifuged at 2150 g for 10 min. Two hundred microliters of RSV-infected Vero cells and 500 ul of HSV-1-infected MRC-5 cells treated with each MBS extract dilution were transferred to a second Vero cell monolayer already cultured in 24-well culture plates for RSV and 6-well culture plates for HSV-1 (Orange Scientific, Europe). Serial 2-fold dilutions of infected Vero cells from the primary plate were prepared in the secondary plates. Each well contained the infected Vero cells from the primary plate, which was exposed to certain extract dilution, plus RPMI-1640 maintenance medium with the final volume of 200 μl/well for RSV and 500 ul/well for HSV-1 except for the first well which contained the infected Vero cells from the primary plate without diluting RPMI-1640 medium. The virus suspensions from the positive control wells of primary plates were transferred to the positive control wells in the secondary plates. All of the plates were incubated for 1.30 h and 1 h for RSV and HSV-1 adsorption, respectively. Then, for RSV, all of the wells’ contents were removed and replaced with 500 μl RPMI-1640 maintenance medium and incubated in a humidified 5 % CO_2_ atmosphere for 48 h at 37 °C. The negative control wells contained cells without virus or the initial extract dilution. The primary and secondary plates’ preparations were repeated three times with four replicates of the extract dilution. After 48 h the virus titer was estimated by endpoint titration method as TCID50/ml see section. For HSV-1, all the wells’ contents were removed and the cells were overlaid with RPMI-1640 culture medium containing 0.5 % agarose type VII. The next steps were carried out as mentioned earlier in virus titration using plaque assay. The primary and secondary plates were repeated three times with four and two replicates to each extract dilution for the primary and secondary plates, respectively, in each run.

### Virucidal activity assay

The virucidal activity assay determines the ability of MBS extract to directly inactivate the extracellular RSV and HSV-1. The viruses were incubated with the non toxic concentrations of extract for different time intervals. Then, the treated virus suspensions were transferred to a ready cell monolayer of virus specific cell line and incubated for 72 h. In other words, cultures were incubated until the wells containing infected cells with untreated virus (positive control) showed complete (100 %) cytopathic effect. In order to compare the antiviral activities of MBS extract with the antiviral activity of the standard drug, the same steps were repeated using the non-cytotoxic concentration of standard drug. The standard drugs were incubated with the viruses for a duration of time equal to that needed by the extracts to cause significant virucidal activity. The cytopathic reduction assay was estimated by recording the level of the virus-induced CPE after the incubation time and was recorded as CPE percentage in comparison with the positive and negative controls, as follows: (0 % CPE), (0-25 % CPE), (25-50 % CPE), (50-75 % CPE) and (75-100 % CPE), [17, 72]. The concentration that reduced 50 % of CPE with respect to control virus was estimated from the plots of the absorbance data obtained from MTT assay. Moreover, in order to confirm the MTT assay results, the monolayer was observed microscopically to estimate virus-induced CPE% as mentioned above [73]. The concentration that reduced 50 % of CPE was also defined as 50 % inhibitory concentration (IC50). IC50 was determined as the lowest concentration of extract that caused 50 % inhibition of the virus-induced CPE. It was calculated using the following formula:

$$ \mathrm{Inhibition}\ \% = \left[\left(\mathrm{O}\mathrm{D}\mathrm{t}\ \hbox{--}\ \mathrm{ODcv}\right)\ /\ \left(\mathrm{OD}\mathrm{cd}\ \hbox{--}\ \mathrm{ODcv}\right) \times 100\right] $$

Where ODt is the test absorbance and ODcv and ODcd are the absorbance of control virus and control cell, respectively [74].

The selective index (SI), which is an important parameter to evaluate the antiviral activity, was calculated from the ratio CC50/IC50 [75]. The assay was repeated three times with four replicates for each test in each run.

### Virucidal activity against RSV and HSV-1

The effects of non cytotoxic concentrations of MBS extract on the extracellular RSV and HSV-1 were investigated by incubating 3.16 × 10^3^ TCID50/ml of RSV and 5.01 × 10^3^ PFU/ml of HSV-1 suspensions in PBS with 2-fold serial dilutions of MBS extract. The MBS stock extract was 441.92 mg/ml for RSV and 273.16 mg/ml for HSV-1 at serial dilutions ranging from 220.96 to 13.81 mg/ml and 136.58 to 8.53 mg/ml, respectively. Using 1.5 ml microtubes, 150 μl of virus suspension were incubated with 150 μl of MBS extract serial dilution in RPMI-1640 maintenance medium. The tubes were incubated in humidified 5 % CO_2_ at 37 °C for zero, 1, and 2 h. Ninety six-well flat bottom tissue culture plates with Vero cell monolayer (1 × 10^5^ cell/well), for RSV, and with MRC-5 cell monolayer (3 × 10^4^ cell/well), for HSV-1, were prepared in advance. The virus suspension with the extract serial dilutions of zero hour time of incubation was directly transferred to the ready tissue culture plates and incubated for 1.30 h to enable virus adsorption. The same procedure was carried out for other microtubes after 1 and 2 h time of incubation. After virus adsorption, the wells’ contents were removed and replaced with 200 μl/well maintenance medium and incubated for 72 h at the same conditions. Then, two hundred microliters of maintenance medium were added to the negative control wells (cells without virus or treatment) and to the positive control wells (cells with virus and without treatment), [76, 77].

The same procedure was repeated on Vero and MRC-5 cell monolayers using the RSV standard drug ‘Ribavirin’ and the HSV-1 standard drug ‘Acyclovir’ except for the following differences: the non cytotoxic 2-fold serial dilutions of Ribavarin and Acyclovir ranged from 2.28 to 0.018 mg/ml and 0.3516 to 0.0027 mg/ml prepared from stock solutions (4.56 mg/ml) and (0.7 mg/ml) in PBS, respectively. RSV was incubated with Ribavirin only for 1 h before the addition to Vero cell monolayer in 96-well flat bottom tissue culture plates while HSV-1 was incubated only for 2 h with Acyclovir before the addition to the ready MRC-5 cell monolayer in 96-well flat bottom tissue culture plates.

### Prophylactic activity assay

In order to evaluate the prophylactic activity of MBS extract on virus specific cells, non cytotoxic extract serial dilutions were incubated with the cells before the virus addition. In order to compare the prophylactic activities of MBS extract with the standard drug of each virus, the standard drugs were incubated with the cells only for duration of time needed by the extract to show significant prophylactic activity. The CPE% together with the concentration that inhibited 50 % of virus-induced CPE or the 50 % inhibitory concentration (IC50) and the (SI) were calculated as mentioned earlier. The assay was repeated three times with four replicates for each well.

### The prophylactic effects of the MBS extract on Pre-RSV-infection vero cell line and Pre-HSV-1 infection MRC-5 cell line

A monolayer of Vero cells (1 × 10^5^ cell/well) and MRC-5 cells (3 × 10^4^ cell/well) in 96-well flat bottom tissue culture plates were treated with 2-fold serial dilutions of MBS extract. The MBS stock extract was, for RSV, 441.92 mg/ml at serial dilutions from 220.96 to 13.81 mg/ml and, for HSV-1, 273.16 mg/ml at serial dilutions from 136.58 to 8.53 mg/ml. The plates were divided into two groups. The first group was incubated with the extract for 30 min and the second group was incubated with the extract for 1 h in humidified 5 % CO_2_ incubator at 37 °C. All of the plates contained extract dilutions as well as RPMI-1640 maintenance medium with the final volume of 200 μl/well. The wells’ contents were removed after the incubation time for each group and the RSV suspension of 3.16 × 10^3^ TCID50/ml and HSV-1 suspension 5.01 × 10^3^ PFU/ml in PBS were added to all the wells except for the negative control wells. The plates were incubated for 1.30 h at the same conditions. After the virus adsorption, the virus suspension was removed and replaced with 200 μl/well of maintenance medium. The positive control wells contained infected cells without treatment. Two hundred microliters of maintenance medium were added to the negative control wells (cells without virus or treatment). All of the plates were incubated for 72 h at the same conditions [78, 79].

The prophylactic activity of the standard RSV drug ‘Ribavirin’ on Vero cell monolayer and the prophylactic activity of HSV-1 standard drug ‘Acyclovir’ on MRC-5 cell monolayer were evaluated using the same procedure described above except for the following differences: the non cytotoxic 2-fold serial dilutions of Ribavarin ranged from 2.28 to 0.018 mg/ml prepared from stock solution of 4.56 mg/ml in PBS while the non cytotoxic 2-fold serial dilutions ranged from 0.3516 to 0.0027 mg/ml prepared from stock solution of 0.7 mg/ml in PBS. The cells were incubated with Ribavirin for 1 h before the addition of RSV to the ready Vero cell monolayer in 96-well flat bottom tissue culture plates while the cells were incubated with Acyclovir for 1 h before the addition of HSV-1 to the ready MRC-5 cell monolayer in 96-well flat bottom tissue culture plates.

### Cytokine production by human embryonic fibroblast cell lines (MRC-5) in response to HSV-1 infection and extract’s treatment

#### The preparation of MRC-5 cells supernatants

The level of antiviral cytokines, IFNβ, TNFα, IL-1, and IL-6, was investigated in four groups of MRC-5 cells. The first group was composed of extract-untreated and HSV-1-non-infected MRC-5 cells (the control cells); the second group was composed of HSV-1-infected cells without extract treatment (infected cells); the third group was composed of extract-treated cells without HSV-1 infection (treated cells); and the fourth group was composed of extract-pretreated and HSV-1-infected cells (treated-infected cells). A number of 3 × 10^4^ cell/well of MRC-5 cells were grown in RPMI-1640 cutlure medium in 96-well flat-bottom tissue culture plates in a humidified 5 % CO_2_ atmosphere for 24 h at 37 °C. For the infected, treated, and treated-infected groups, the procedures used were the same to that described in section (3.2.9.2) for prophylaxis of MRC-5 cells against HSV-1 using the extracts’ IC50 values of IVR protocols which are 12.72 mg/ml. MBS extract was incubated in triplicates with MRC-5 cells for the optimal time found earlier, namely 1 h. The final volume of wells was 200 μl/well. A triplicate of negative control wells contained cells with RPMI-1640 maintenance medium only with final volume of 200 μl/well. Afterwards, the supernatants from all the wells were removed and centrifuged at 1000 g for 10 min to be ready for the ELISA technique [80].

### Sandwich Enzyme-Linked Immunosorbent Assay (ELISA)

The concentration of antiviral cytokines in the supernatant of culture medium of MRC-5 cells was determined by sandwich ELISA. MRC-5 cell line was chosen because MRC-5 cells are human cells able to produce many human cytokines including the antiviral cytokines, TNFα, IFNβ, IL-1, and IL-6. On the other hand, Vero cell line which was used in the antiviral assays done earlier on RSV, was not used in measuring the antiviral cytokines because Vero cells are not of human origin and IFNβ-deficient cells.

The measurement of human TNFα using immunometric assay (EIA) kits (Cayman, USA) was done according to the kit’s instructions. In order to prepare the standard curve, the standard of TNFα was reconstituted in EIA buffer. The standard dilutions must be present in the same biological fluid “matrix” as that of the sample to accurately assay unpurified samples. The standard was diluted in the sample matrix (the IC50 of MBS extract in RPMI-1640 culture medium) excluded from the target cytokine, TNFα. The standard dilutions, in triplicates, were prepared as 2-fold serial dilutions ranged from 250 to 3.9 pg/ml in eight tubes of 15 ml capacity (Orange Scientific, Europe); the 8th tube containing only the sample matrix with zero concentration of the standard. One hundred microliters of the standard or the samples were added to the microtiter plate which was supplied with the kit. The microtiter plate was already coated with monoclonal antibodies specific for TNFα. The samples were assayed in triplicates. One hundred microliters of Acetylcholinesterase: TNFα Fab’ conjugate were added to the wells of the standards and the samples. The plates were covered with plastic films and incubated overnight at 4 °C. The quantification of the conjugate was achieved by measuring its acetylcholinesterase activity with freshly prepared Ellman’s reagent (acetylcholine and 5,5’-dithio-*bis*-(2-nitrobenzoic acid). All of the wells were rinsed 5–6 times with washing buffer after removing their contents. Two hundred microliters of Ellman’s reagent were added to all of the wells. The plate was covered with plastic film and incubated for six hours at 25 °C in shaker incubator. Blank wells contained only Ellman’s reagent added to the triplicate wells of the 8th standard (to subtract the color effect). Later, the absorbance (OD) was measured at 412 nm (reference 690 nm) using a 96-well plate ELISA reader. The samples’ concentrations were determined by extrapolating OD values of TNFα to the generated linear standard curve (the average absorbance on the vertical axis versus the corresponding standard concentration on the horizontal axis).

For the measurement of the concentration of IFNβ, IL-1, and IL-6 in the supernatant of MRC-5 cell culture medium, an ELISA kit from (abcam, USA) was used. IFNβ, IL-1, and IL-6 standards were freshly prepared by reconstituting in the standard diluent buffer to give a stock concentration of 400 pg/ml. Two hundred microliters of this stock was added in triplicate to the already coated microtiter plates provided by the kit. Each plate was coated with monoclonal IFNβ, IL-1, or IL-6 specific antibodies. From stock wells serial 2-fold dilutions, in triplicates, of IFNβ, IL-1, and IL-6 standards were prepared by diluting the standard in the sample matrix which composed of the IC50 of MBS extract in RPMI-1640 medium, excluded from the target cytokines. IFNβ, IL-1, and IL-6 standards’ concentrations ranged from 400 to 12.5 pg/ml; and the last standard contained zero concentration of IFNβ, IL-1, and IL-6 standards in sample matrix. The prepared samples (100 μl/well) were added, in triplicates. Then, 50 μl of freshly prepared biotinylated anti- IFNβ, IL-1, or IL-6 antibodies, diluted in biotinylated antibody diluents, were added to all of the wells. The plates were covered with plastic films and incubated two hours at 25 °C. Later, all the wells’ contents were removed and the wells were washed for two times with washing buffer. One hundred microliter of freshly prepared Streptavidin-horse radish peroxidase (Streptavidin-HP), diluted in HP diluent, were added to all wells. The plates were covered and incubated for 30 min at 25 °C. Two washes with washing buffer were done to rinse all the wells. One hundred microliters of freshly prepared substrate solution; chromogen TMB (5-thio-2-nitrobenzoic acid) were added to the wells and incubated in dark for 20 min. The reaction was terminated by adding H_2_SO_4_, a stopping reagent. The blanks, in triplicates, were composed of the substrate chromogen solution along with the stopping reagent added to the zero standard wells. A colored product was formed in proportion to the level of IFNβ, IL-1, and IL-6 cytokines in the tested supernatants. The absorbance was measured at 450 nm (reference 620 nm) by ELISA reader. The samples’ concentrations were determined by extrapolating OD values of IFNβ, IL-1, or IL-6 samples to the generated linear standard curve.

### Data analysis

Data are presented as mean ± 2SE from three independent experiments. The selectivity index (SI) was determined by the ratio of CC50 to IC50. The effect of the tested MBS extract on the inhibition of viral replication was evaluated by SPSS software version (12.0.0.2). The statistically different effects of the extract on the inhibition of RSV or HSV-1 replication were compared with the control groups using the Student’s *t*-test. Differences were considered significant at *P* <0.05.

## Abbreviations

MBS: Mung bean sprout
RSV: Respiratory syncytial virus
HSV-1: Herpes simplex virus-1
RF: Reduction factor
CPE: Cytopathic effect
MTT: Microculture tetrazolium assay
EPTT: End point titration technique
DVI: Direct Virus Inactivation
IVR: Inhibition of Virus Replication
CC50: Cytotoxic concentration 50
IC50: Inhibitory concentration 50
SI: Selective index
IFNβ: Interferon beta
IL-1: Interleukin 1
IL-6: Interleukin 6
TNFα: Tumor necrosis factor alpha
